# Effect of Acute Psychological Stress on Speed Perception: An Event-Related Potential Study

**DOI:** 10.3390/brainsci13030423

**Published:** 2023-02-28

**Authors:** Jifu Wang, Lin Yu, Feng Ding, Changzhu Qi

**Affiliations:** 1 College of Education and Physical Education, Yangtze University, Jingzhou 434023, China; 2 Neurocognition and Action-Biomechanics Research Group, Faculty of Psychology and Sports Science, Bielefeld University, 33501 Bielefeld, Germany; 3 Department of Psychology, Wuhan Institute of Physical Education, Wuhan 430079, China

**Keywords:** ERPs, acute psychological stress, speed perception, light dots expansion task

## Abstract

The present study tested the intrinsic ERP features of the effects of acute psychological stress on speed perception. A mental arithmetic task was used to induce acute psychological stress, and the light spot task was used to evaluate speed perception. Compared with judgments in the constant speed and uniform acceleration motion, judgments in the uniform deceleration motion were made more quickly and with higher accuracy; attention control was higher and peaked later; and there was longer N2 peak latency, larger N2 peak amplitude, and lower mean amplitude of the late negative slow wave (SW). Under stress, the reaction time was significantly shorter. The N2 peak amplitude and SW mean amplitude were significantly higher, attention control was higher and appeared earlier, and there was a greater investment of cognitive resources. The type of movement and evoked stress also interacted to predict behavioral and ERP measures. Under acute stress, judgments made in the uniform deceleration motion condition elicited lower N2 peak latency, higher attention control, and later peak attention. The results suggest that judgments of the speed of decelerating motion require a lower investment of cognitive resources than judgments of other kinds of motion, especially under acute stress. These findings are best interpreted in terms of the interaction of arousal and attention.

## 1. Introduction

The direction and speed of moving objects are often critical for survival [1]. Speed perception plays an important role in driving, walking, running, and other activities such as sports [2]. Previous studies showed that speed perception might be influenced by multiple factors [3]. These factors that affect the speed perception include movement direction, stimulus contrast [4,5,6], background interference [3], movement form [7], emotional state [8], and cognitive style [9]. With regard to emotional state, no studies to date have tested the effect of acute psychological stress on speed perception. In the current study, we tested the effect of acute psychological stress on speed perception, with a focus on both how quickly and accurately people judge the tasks of speed stimulation and ERP activity while people make these judgments. This study is an experimental study, which can provide empirical support for the research of speed perception.

Speed perception is part of motion perception and refers to the judgment of the speed of moving objects. The dot stimulation of movement is one of the main research paradigms of speed perception [10,11]. Research found that when rotating and expanding random dot patterns with identical dot speeds are compared, the dots in expanding patterns appear to move faster [7]. Bex et al. (1999) found that motion aftereffects for expanding stimuli were stronger than for translating stimuli [12]. This is the reason why we choose the extended patterns of dot stimulation. How does stress affect speed perception? This is the purpose of this study. Metacognition is involved in the completion of speed perception tasks. Metacognition improves superior cognitive abilities which are essential for stress regulation [13]. Metacognition can strengthen the brain regions that control stress and weaken the structures that enhance stress [13]. So, the metacognitive ability makes a link between speed perception and stress.

Acute stress can be defined as a non-specific physiological response to any threatening stimulus that exceeds the body’s endurance in a very short time [14]. This non-specific response involves the secretion of cortisol and catecholamines, which have wide-ranging effects on the body. Stress affects cognitive functions by increasing dopaminergic, noradrenergic, or glucocorticoid-mediated signaling [15,16]. These effects may include changes in attention, which is directly related to the information processing in perception [17]. For example, a previous study reported that acute stress may increase alertness levels and promote attention control in the process of selective attention [18]. Another study found that acute stress could improve behavioral performance and enhance the investment of attention resources on the perception of motion in depth tasks [19]. In addition, acute stress is inclined to promote cognitive processes in easy tasks and in those with low cognitive load [20]. Based on this evidence, acute psychological stress appears to have an impact on the cognitive process, but whether it affects speed perception is still unknown.

There are mixed perspectives on the question of whether stress is related to the perception of speed. On the one hand, there are theories that assert that emotional processing and cognitive processing are interactive. For example, cognitive resource limitation theory assumes that people can only allocate limited cognitive resources within a certain period of time [21]. Similarly, the dual competition model predicts that when cognitive processing and emotional processing occur at the same time, they will compete for limited cognitive resources [22]. These theories predict that acute psychological stress interferes with cognitive functioning, including the perception of speed. On the other hand, there are theories that predict that an appropriate level of emotional stimulation would have a positive effect on cognitive processing. For example, the Yerkes–Dodson law (also known as the inverted “U” hypothesis) predicts that task performance will be better under moderate arousal than under low or high arousal [23]. Similarly, according to neuromotor interference theory, interference of emotion processing does not necessarily cause a decline in task performance [24]. On the basis of these models, does acute psychological stress affect speed perception? In addition, what is the mechanism behind this influence? In this study, the effect of acute psychological stress on speed perception and its neural mechanism was investigated by event-related potential (ERP) for the first time.

The brain area MT (Middle Temporal) is not only a key structure in the analysis of object speed but also in the analysis of the depth perception and direction of motion [1,25]. Speed tuning in MT could be primarily inherited from V1 [26]. Speed and direction are separated from other attributes of motion (such as spatial frequency) in the early stages of vision [27]. Patients with MT lesions cannot perceive movement stimulation with a speed exceeding 6°/s [28]. Regarding the ERP component of speed perception, previous research used the electroencephalogram (EEG) method to explore differences in speed perception across three driving speeds (25, 50, and 75 km/h) and found activity in the N2 component in the middle temporal area. There were significant differences in the N2 peak latency and peak amplitude among the three speeds [29]. ERP components related to speed perception also include P1 and slow wave potentials (SW). The P1 component of the temporo-occipital region was related to visual information processing of the speed perception stimulus [30]. SW mean amplitudes of the frontal area were easily affected by memory load [31].

In the current study, we used the modified Montreal imaging stress task (MIST) to induce acute psychological stress in the laboratory [32]. The Emotional State Evaluation Scale was used to evaluate the effect of stress induction before and after the experiment [33]. We then assessed behavioral indicators of speed perception (response time and accuracy in identifying the type of motion) and EEG activity in the P1 component, N2 component, and SW mean amplitude of speed perception under stress and no stress. The tasks of speed perception included constant speed (CS), uniform acceleration (UA), and uniform deceleration (UD). Allen et al. (2022) found that higher psychological arousal was induced by acute stress [34]. Time perception was affected by psychological arousal [35]. Speed perception was a perception based on space perception and time perception. So, we hypothesized that under acute psychological stress, people process information faster in speed perception. Subsequently, people will show shorter ERP latency and higher amplitude at P1, N2, and SW. Through the research of this experiment, people can deeply realize that acute psychological stress is not all harmful to our bodies.

## 2. Methods

### 2.1. Participants

A priori power analysis was conducted using G*power 3.1.9 [36] to determine the necessary sample size to detect a medium effect size (d = 0.25) with α = 0.05 and power = 0.80 using repeated measures analysis of variance. Based on the prior power analysis in G*Power 3.1.9, the results showed that 19 participants were required. Additionally, according to the previous study [20], our sample was set as *N* = 24 (12 males). More sample size can increase the reliability of the experimental results. The mean age of participants was 19.54 ± 1.22 (M ± SD) years. All subjects had normal or corrected-to-normal vision, and all were right-handed. All subjects provided written informed consent and received 60 RMB after the experiment. This study was approved by the Institutional Research Ethics Committee of the first author’s affiliated university.

The inclusion of participants needed to meet the following criteria: (1) no depression and anxiety; (2) stable emotional state; (3) good spatial thinking ability. So, the Baker Depression Questionnaire [37], the Emotional State Evaluation Scale [33], the State-Trait Anxiety Inventory [38], and the Mosaic Pattern Test [39] were used to screen 24 undergraduate students’ depression, excitement state, and spatial thinking ability. The purpose of the Mosaic Pattern Test was to ensure that the cognitive style of all participants was field independent. A statistical analysis of the scale scores found that none of the 24 subjects was in negative emotional state or excessive excitement when they participated in the experiment. The spatial thinking ability of all subjects was basically at the same level (M ± SD = 116.88 ± 4.79, the total score was 122).

### 2.2. Design and Stimuli

This study employed a 2 (stress levels: control vs. stress) × 3 (speed types: UA, UD vs. CS) within-subjects experimental design. Acute psychological stress was induced by a set of multiplication formulas containing 120 arithmetic expressions (e.g., 2.16 × 4.78). Subjects were asked to judge the relationship between multiplication results and 10. They were instructed to press j on the keyboard if they thought the multiplied result was larger than 10, and to press f otherwise. In previous research, the modified MIST task has been shown to produce lower positive affect and higher self-reported state anxiety [18]. Combined with the results of previous studies [18,19,40], the modified MIST task had a stress-inducing effect.

Drawing on Hietanen et al. (2008) research paradigm [11], stimulus materials were composed of scattered dots of light spreading from the center to the surrounding area, viewed on a screen. These materials were made using Autodesk 3dsMax 2010 as videos. All videos were formatted in WMV (Windows Media Video), and the resolution was set as 1024 × 768. The video was displayed on a 19-inch monitor about 70 cm ahead of the participants. The scattered dots were white, and the dot diameter was 0.04°. There were 12 flying directions for the dots, and the angle between directions was 30°. The dots’ speed was presented in three conditions. Under the condition of uniform acceleration, the initial velocity of the dots was 0°/s, and the acceleration was 0.03°/s^2^. Under the condition of uniform deceleration, the initial velocity of the dots was the speed at which the dots disappeared from the boundary under the condition of uniform acceleration (0.06°/s), and the acceleration was −0.03°/s^2^. Under the condition of constant speed, the constant velocity of the dots was the average velocity (0.03°/s) from the appearance of the dots to the disappearance of the boundary under the condition of uniform acceleration. The background of the video was black, and the length of each video was 2000 ms. The scattered dots moved outward from the initial visual angle of 0.32° to the final visual angle of 13.85° in the retina. The speed at which dots moved in the retina was 6.93°/s. One second of video included 50 frames. The running time of the outermost dots was 100 frames (the dots disappeared after 100 frames), followed by 80 frames, 60 frames, 40 frames, and 20 frames. The dot appeared in the specified number of frames and then disappeared. The experimental stimulus materials were presented by E-prime 2.0 software (Psychology Software Tools, Inc., Pittsburgh, PA, USA). To avoid the influence of luminance on speed perception [41], the luminance of laboratory was constant.

Multi-channel physiological recorder (Mind Ware, OH, USA) was used to measure heart rate (HR) of participants. The ECG channel was selected in Biolab, and the sampling rate was 500 Hz. The SDNN (standard deviation of normal to normal) was recorded using the Mind Ware. The SDNN could be used to indirectly reflect the size of vagus nerve activity.

### 2.3. Procedure

Participants filled out the consent forms and completed the screening measures. Participants were asked to sit up straight during the experiment, keep their eyes at a distance of about 70 cm from the center of the screen, and try to avoid gross body movements such as swinging their heads or legs. Participants were then asked to carefully observe a relaxing picture presented on the screen and to imagine themselves immersed in it (adjusting their breathing to relax their heart rate for one minute). The main purpose of this relaxation process was to prepare the participants for the experiment. There was then a practice stage (12 trials) in which participants received feedback about whether the judgment of speed was correct.

**Figure 1:**
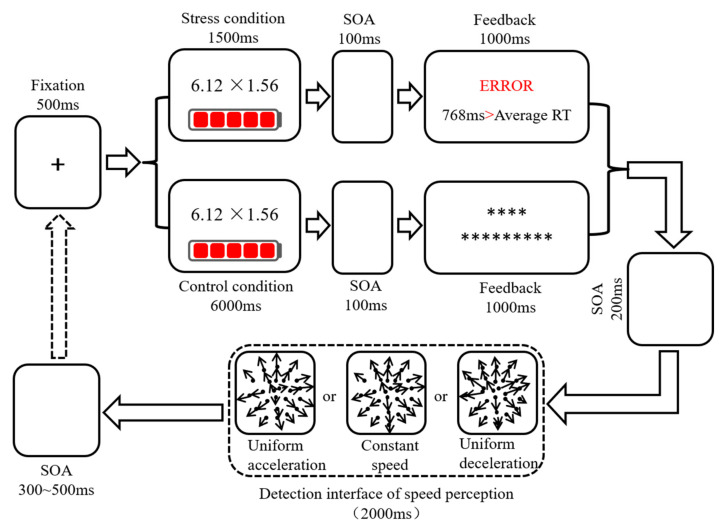
Schematic diagram of the experimental procedure of stress-inducing and speed perception. “*” was the content of feedback.

Using the modified MIST paradigm to induce acute stress, participants completed a mental arithmetic task and received two types of feedback. Under the stress condition, the participant received feedback about the accuracy of their response and a comparison between their reaction time (RT) and that of the average person. The RTs of average person were about 700 random numbers. Under non-stress state, the feedback was only a set of asterisks (Figure 1). The mental arithmetic items in the practice trials were different from those in the formal experiment. The formal experimental stage included two blocks: stress condition (120 trials) and control condition (120 trials). Uniform acceleration, uniform deceleration, and constant speed each included 40 trials, and each trial was presented randomly. There was a practice block before the stress and control conditions. In order to eliminate the influence of item differences on the experimental results, the mental arithmetic items selected for the control and stress conditions were consistent. In order to avoid experimental errors caused by the sequence effect, the study adopted an ABBA design to balance the trials.

To avoid interaction between the two conditions, participants rested for 10 min between the two blocks. Before the formal experiment, the subjects were asked to adjust their emotional state by looking at the presumably relaxing scene (one minute). The instructions for the speed perception tasks had three parts. “If all the dots were uniformly accelerated from the inside to the outside, please press j on the keyboard with your right index finger. If all the dots were decelerating from the inside to the outside, please press f on the keyboard with your left index finger. If all the dots were moving at a constant speed from the inside to the outside, please press the ‘space’ key with your right thumb.” After each item, the feedback interface showed whether the subject’s response was correct or not.

### 2.4. Behavioral Data Recording and Analysis

Behavioral data were collected by E-prime 2.0. The SPSS17.0 software (International Business Machines Corporation, New York, NY, USA) generated descriptive statistics for reaction time and accuracy under stress and control conditions. A 2 (stress/control) ×2 (male/female) × 3 (CS/UA/UD) repeated measurement analysis of variance was conducted with reaction time to identify the different effects among the conditions. Differences in accuracy (percentage) were tested with chi-square analysis.

### 2.5. Electrophysiological Recording and Analysis

Brain activations were recorded using a 64-channel EEG amplifier (Brain Products, Munich, Germany) with BrainVision Recorder software. Sixty-four Ag/AgCl electrodes were placed according to the international 10-10 system. The impedance of all electrode sites was under 5 kΩ. The electrode AFz was selected as the recording ground, and the electrode FCz was used as the recording reference. Two extra electrodes were used to record the electrooculography (EOG). They were horizontal EOG and vertical EOG. All signals were filtered from 0.01 to 100 Hz online and sampled at 1000 Hz before digitization.

EEG raw signals were processed offline with the BrainVision Analyzer software (version 2.0). Signals were first re-referenced from FCz to average reference, and then IIR filtered from 0.01 to 35 Hz. Independent component analysis (ICA) was applied for the EOG correction [42]. Semiautomatic mode of ocular correction was used to eliminate the EOG component in the blink interval. The mean slope algorithm was adopted in the detection algorithm of blink detection algorithm. The length and bad interval free for ICA interval were both 50 s. A 1200 ms epoch (time-locked to the onset of the scattered dots) was selected for ERP analysis, and the period of the first 200 ms was used for baseline correction. Trials with artifacts (peak-to-peak amplitude larger than 80 μV) were removed from the grand average. In this study, the average number of superpositions in each experimental trial was 36. This result met the minimum requirement of 30 times proposed in previous study [43].

According to the results of previous studies [29,44] and the total waveform of ERPs from this study, we focused on P1, N2, and SW. The PO3, PO7, POz, PO8, and PO4 (80–200 ms) were selected as the electrode sites for the analyses of the P1 peak latency and amplitude. The CP5, CP6, P7, and P8 (150–250 ms) sites were selected for analysis of the N2 peak latency and amplitude. The Fz, F1, and F2 (400–800 ms) sites were chosen as electrode sites for analyses of SW mean amplitude. A repeated measures ANOVA was used to analyze the results obtained in the experimental task. The Greenhouse–Geisser correction was used to adjust for sphericity violations when the degree of freedom was more than one [40].

## 3. Results

### 3.1. Stress Manipulation Checking

**Figure 2:**
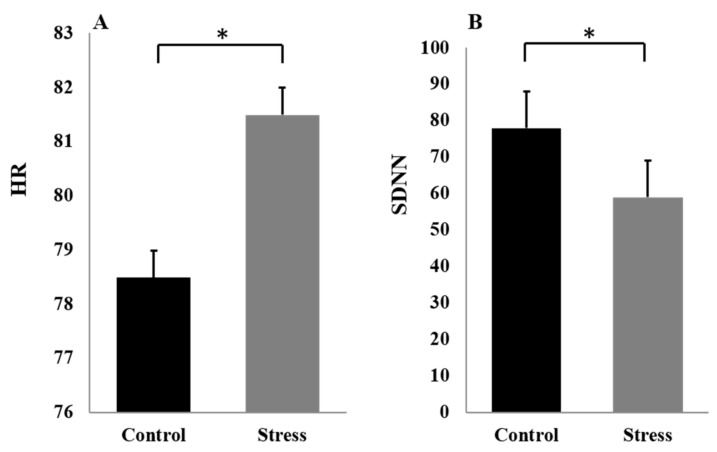
HR (**A**) and SDNN (**B**) at different stress levels. The error bars indicate the *SEM*. “*”means “ *p* < 0.05”.

The HR was higher stress versus control, t (23) = 2.56, *p* < 0.05 (see Figure 2A). The SDNN was lower control versus stress, t (23) = −2.29, *p* < 0.05 (see Figure 2B). On the mental arithmetic task, shorter RT, t (23) = −6.87, *p* < 0.01, and lower accuracy, $\chi^{2}$ = 9.89, *p* < 0.01, were found in the stress condition versus the control condition. Combined with the results of previous studies [18,19,40], it can be concluded that the improved MIST task had a stress-inducing effect.

### 3.2. Behavioral Data

#### 3.2.1. Reaction Time

**Figure 3:**
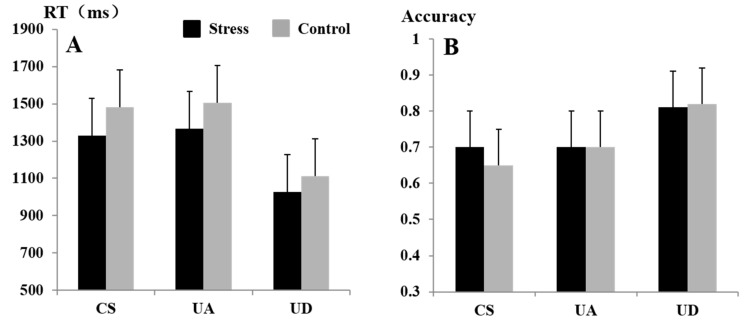
RT (**A**) and accuracy (**B**) at different stress levels and speed types. The error bars indicate the *SEM*.

The findings on the RT of speed perception tasks showed that the main effects of stress levels, F(1, 22) = 12.37, *p* < 0.01, $\eta_{p}^{2}$ = 0.35, were significant. $M_{\mathrm{stress}}$ = 1240.98 ms < $M_{\mathrm{control}}$ = 1366.11 ms (see Figure 3A). The main effects of gender, F(1, 22) = 0.17, *p* > 0.05, were not significant. The main effects of speed types, F(1.49, 32.82) = 55.81, *p* < 0.01, $\eta_{p}^{2}\;$ = 0.71, were significant. Post hoc testing showed that the difference in RT between CS and UA was not significant (*p* > 0.05). The difference between CS and UD was significant (*p* < 0.01), and the difference between UA and UD was significant (*p* < 0.01). These comparisons showed that the reaction speed was faster under the UD condition than that of the CS and UA conditions. The interaction effects between stress levels and speed types in predicting reaction time, F(1.33, 29.33) = 1.67, *p* > 0.05, $\eta_{p}^{2}$ = 0.07, were not significant.

#### 3.2.2. Accuracy

A chi-square test was conducted to examine whether the % accuracy score varied depending on stress levels and speed types. The % accuracy scores did not differ according to stress levels, $\chi^{2}$ = 0.31, df = 1, *p* > 0.05. The % accuracy scores did not differ according to gender, $\chi^{2}$ = 3.43, df = 1, *p* > 0.05. However, % accuracy scores did vary by speed types, $\chi^{2}$ = 28.81, df = 2, *p* < 0.01. The interaction effects between speed types and stress levels, $\chi^{2}$ = 1.39, df = 2, *p* > 0.05, were not significant. The accuracy results are presented in Figure 3B. The participants were significantly more accurate in identifying UD compared with CS and UA.

### 3.3. Electrophysiological Data

#### 3.3.1. P1 Peak Latency

**Figure 4:**
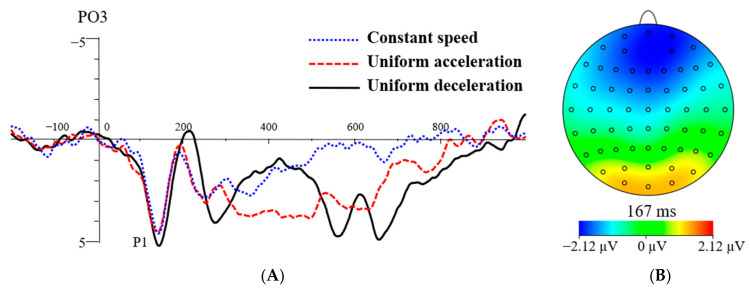
The grand average waveform for P1 at speed types of CS, UA, and UD (**A**). The topographic map illustrates the difference in P1 between the UD and UA (**B**).

The findings on the P1 peak latency showed that the main effects of stress levels, F(1, 23) = 0.73, *p* > 0.05, $\eta_{p}^{2}$ = 0.03, were not significant. The main effects of electrode sites, F(2.11, 48.58) = 0.70, *p* > 0.05, $\eta_{p}^{2}$ = 0.03, were not significant. The main effects of speed types, F(2, 46) = 7.07, *p* < 0.01$,\;\eta_{p}^{2}$ = 0.24, were significant. The P1 peak latencies were 137.28 ms for UA, 145.88 ms for UD, and 142.11 ms for CS (see Table 1). Post hoc testing showed that the peak latency of P1 was significantly shorter in the UA condition than that of the UD condition (*p* < 0.05), and there was no significant difference between the other two pairs. The interaction effects between electrode sites and stress levels, F(2.71, 62.43) = 0.70, *p* > 0.05, $\eta_{p}^{2}$ = 0.03, were not significant. There was no significant difference in the other pairwise interactions (*p* > 0.05). From the total average waveform of the P1 component, it can be concluded that the peak latency of P1 was higher in the UD condition than that of the UA and CS conditions (see Figure 4A).

#### 3.3.2. P1 Peak Amplitude

A similar repeated measures ANOVA of P1 peak amplitude showed a significant main effect of stress levels, F(1, 23) = 1.00, *p* > 0.05$,\;\eta_{p}^{2}\;$ = 0.04. The main effects of electrode site, F(2.16, 49.61) = 3.28, *p* < 0.05, $\eta_{p}^{2}$ = 0.13, were significant. The P1 peak amplitudes of the five electrode sites were 6.68 μV for PO3, 6.67 μV for PO7, 5.61 μV for POz, 7.03 μV for PO4, 6.03 μV for PO8. Post hoc tests showed that the peak amplitude of P1 was significantly higher at PO3 than at POz (*p* < 0.01), and significantly lower at POz than at PO4 (*p* < 0.01). The other pairwise comparisons were not significant (*p* > 0.05). The main effects of speed type on P1 peak amplitude, F(2, 46) = 2.00, *p* > 0.05$,\;\eta_{p}^{2}\;$ = 0.08, were not significant. There was no significant difference in the two-way interaction of the three independent variables (*p* > 0.05).

#### 3.3.3. N2 Peak Latency

**Figure 5:**
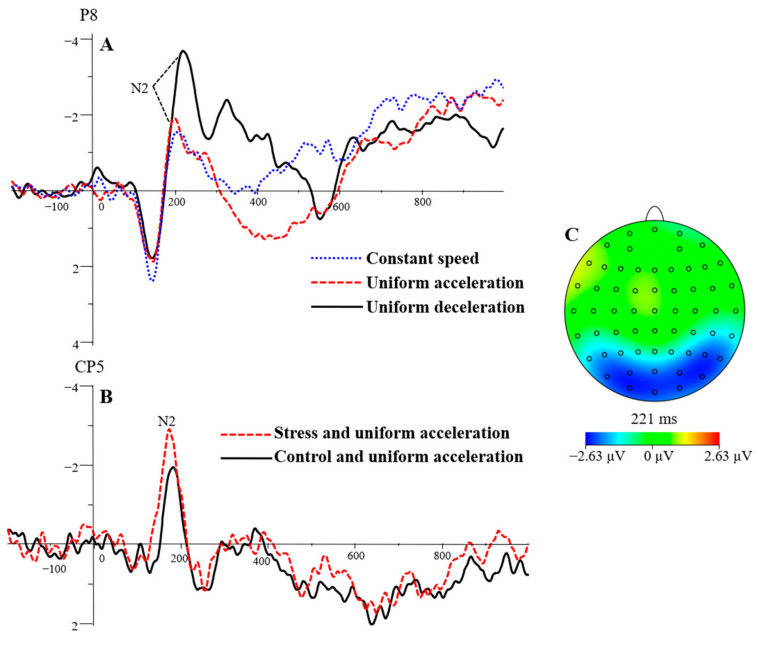
The grand average waveform for N2 at speed types (**A**) and stress levels (**B**). The differences between the UD and UA were shown in the topographic map (**C**).

The main effects of electrode sites, F(3, 69) = 37.89, *p* < 0.01, $\eta_{p}^{2}$ = 0.08, were significant. The N2 peak latency of the four electrode sites was 179.56 ms for CP5, 205.26 ms for P7, 190.38 ms for CP6, and 208.54 ms for P8. The N2 latency was significantly shorter at CP5 than at P7, CP6, and P8 (*p* < 0.01); significantly longer at P7 than at CP6 (*p* < 0.01); and significantly shorter at CP6 than at (*p* < 0.01). The main effects of stress levels, F(1, 23) = 1.61, *p* > 0.05, $\eta_{p}^{2}$ = 0.07, were not significant. $M_{\mathrm{stress}}$ = 194.69 ms, $M_{\mathrm{control}}\;$ = 197.18 ms. The main effects of speed types, F(2, 46) = 9.55, *p* < 0.01, $\eta_{p}^{2}$ = 0.29, were significant. The N2 latencies of the three speed types were 191.71 ms for uniform acceleration, 201.45 ms for uniform deceleration, and 194.65 ms for constant speed (see Table 1). Post hoc testing showed that the N2 latency evoked by uniform acceleration was significantly shorter than that evoked by uniform deceleration (*p* < 0.01). The N2 latency evoked by constant speed was significantly shorter than that evoked by uniform deceleration (*p* = 0.05). The difference in N2 latency between the uniform acceleration and constant speed conditions was not significant (*p* > 0.05). See Figure 5A.

The two-way interactions were as follows. The interaction effects between stress levels and electrode sites on N2 peak latency, F(3, 69) = 1.24, *p* > 0.05, $\eta_{p}^{2}$ = 0.05, were not significant. The interaction between electrode site and speed type on N2 peak latency was significant, F(6, 138) = 4.20, *p* < 0.01, $\eta_{p}^{2}$ = 0.15. Simple effect analysis found that the N2 latencies at CP5 and CP6 were significantly shorter than those at P7 and P8 under the uniform acceleration and uniform deceleration conditions (*p* < 0.01). The N2 latency was significantly higher at P7, CP6, and P8 than at CP5 (*p* < 0.01), and significantly higher at P8 than at CP6 (*p* < 0.01) under CS. The interaction effects between speed types and stress levels, F(1.43, 32.99) = 1.58, *p* > 0.05, $\eta_{p}^{2}$ = 0.06, were not significant. The three-way interaction effects among speed types, stress levels, and electrode sites, F(6, 138) = 3.40, *p* < 0.01$,\;\eta_{p}^{2}$ = 0.13, were significant. Simple effect analysis showed that the peak latency of N2 was significantly shorter in the stress condition than in the control condition at CP5 in UA condition or at P8 in CS conditions (*p* < 0.05). Within the stress condition, the peak latency of N2 was significantly shorter at CP5 than at P7 and P8 in the UA and UD conditions (*p* < 0.01), and significantly longer at P8 than at CP6 (*p* < 0.01). Within the control condition (see Figure 5B), the N2 peak latency was significantly shorter at CP5 than at P7 and P8 (*p* < 0.05) and significantly longer at P8 than at CP6 (*p* < 0.05) in the UA; at CP5 it was significantly longer in the UA than in the UD and CS conditions (*p* < 0.05). Within the stress condition, the peak latency of N2 was significantly shorter in the UA at P7 than in the UD and CS conditions (*p* < 0.05); the peak latency of N2 was significantly longer in the UD at P8 than in the CS condition (*p* < 0.01). Within the control condition, the peak latency of N2 was significantly shorter in the UA at P7 than in the UD condition (*p* < 0.05); the peak latency of N2 was significantly longer in the UD and CS conditions at P8 than in the UA (*p* < 0.05).

#### 3.3.4. N2 Peak Amplitude

The main effects of electrode sites, F(1.78, 40.97) = 3.45, *p* < 0.05, $\eta_{p}^{2}$ = 0.13, were significant. The N2 peak amplitudes at the four electrode sites were −4.07 μV for CP5, −3.10 μV for P7, −4.16 μV for CP6, and −4.16 μV for P8. The peak amplitude of N2 was significantly higher at CP5 than at P7 (*p* < 0.01). There was no significant difference between the other two electrode sites (*p* > 0.05). The main effects of stress levels, F(1, 23) = 0.70, *p* > 0.05, $\eta_{p}^{2}$ = 0.03, were not significant.${\;M}_{\mathrm{stress}}$ = −3.99 μV, $M_{\mathrm{control}}\;$ = −3.73 μV. The main effects of speed types, F(2, 46) = 13.14, *p* < 0.01, $\eta_{p}^{2}$ = 0.36, were significant. The N2 peak amplitudes of the three speed types were −3.39 μV for uniform acceleration, −4.54 μV for uniform deceleration, and −3.65 μV for constant speed. The N2 peak amplitude was significantly higher in the UD than in the UA and CS (*p* < 0.01). There was no significant difference between the UA and CS (*p* > 0.05).

There were significant interaction effects between electrode sites and stress levels, F (2.22, 51.14) = 4.83, *p* = 0.01, $\eta_{p}^{2}$ = 0.17. Simple effect analysis found that the N2 peak amplitude at CP5 in the control condition was significantly lower than in the stress condition (*p* < 0.05), see Figure 5A. In the stress condition, the peak amplitude of N2 was significantly higher at CP5 than at P7 (*p* < 0.01). The interaction effects of electrode sites and speed types, F (3.52, 80.93) = 4.75, *p* < 0.01, $\eta_{p}^{2}$ = 0.17, were not significant. Simple effect analysis found that the N2 peak amplitude was significantly higher in the UD than in the UA and CS at P7 and P8 electrode sites (*p* < 0.01), see Figure 5B. The interaction effects between speed types and stress levels, F(2, 46) = 0.02, *p* > 0.05, $\eta_{p}^{2}$ = 0.001, were not significant. The three-way interaction effects among speed types, stress levels, and electrode sites, F(6, 138) = 0.54, *p* > 0.05, $\eta_{p}^{2}$ = 0.02, were not significant.

#### 3.3.5. SW Mean Amplitude

**Figure 6:**
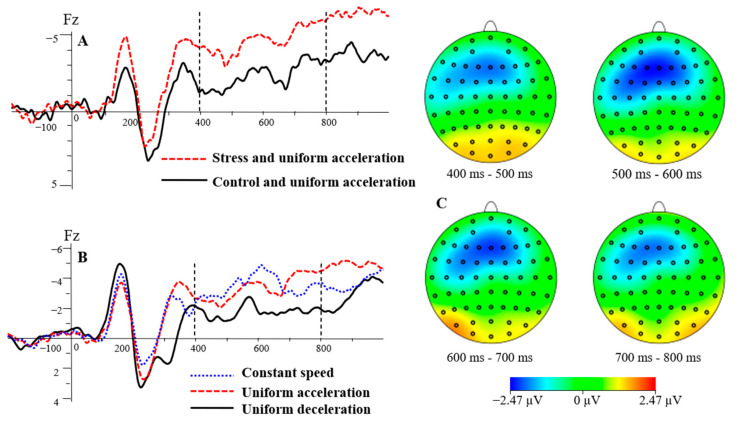
The grand average waveform for SW at stress levels and speed types (**A**,**B**). The differences between the stress and control conditions in the UA condition were shown in the topographic map (**C**).

The main effects of electrode sites, F(2, 46) = 4.42, *p* < 0.05,$\;\eta_{p}^{2}$ = 0.16, were significant. The SW mean amplitude of the three electrode sites was −1.70 μV for Fz, −1.19 μV for F1, and −1.68 μV for F2. The SW mean amplitude was significantly higher at Fz and F2 than at F1 (*p* < 0.05). The main effects of stress levels, F(1, 23) = 8.62, *p* < 0.01, $\eta_{p}^{2}$ = 0.27, were significant. $M_{\mathrm{stress}}$ = −2.53 μV, $M_{\mathrm{control}}\;$ = −0.51 μV. The SW mean amplitude was significantly lower in the control condition than in the stress condition (*p* < 0.01), see Figure 6A. The main effects of speed types, F(1.56, 35.97) = 10.40, *p* < 0.01, $\eta_{p}^{2}$ = 0.31, were significant. The SW mean amplitudes for the three speed types were −1.81 μV for uniform acceleration, −0.10 μV for uniform deceleration, and −2.56 μV for constant speed. The SW mean amplitude was significantly lower in the UD condition than in the UA and CS conditions (*p* < 0.05), and there was no significant difference in the other pairwise comparison (*p* > 0.05). The mean amplitude of SW was significantly lower in the UD condition than in the UA and CS conditions, see Figure 6B. There was no significant difference in the two-way interaction of the three independent variables (*p* > 0.05).

## 4. Discussion

We tested the hypothesis that there was a difference between the control and stress conditions in behavioral performance and EEG activity of the speed perception and give the basic idea about stress and judgments of movement. As expected, there was a shorter RT in the speed perception task under acute psychological stress than under control conditions. The peak amplitude of N2 and the SW mean amplitude during the speed perception task under control conditions were both lower than those under stress. The peak latency of N2 under the control condition was longer than that of the stress condition when subjects were judging the speed of UA and CS. The following will be discussed from the behavioral and ERP results.

The reaction time in making judgments about speed was shorter under stress than that of the control condition. These results are consistent with Dambacher and Hübner (2014) finding that the range of attention became narrower under time pressure, resulting in lower efficiency in perceptual processing, evident in lower accuracy [45]. Bertsch et al. (2011) also found that there was a lower accuracy and faster perception processing speed under stress [46]. These studies, along with the current results, suggest that people’s level of body alertness and self-defense awareness increase under stress, showing a faster perceptual response speed. Speed perception was a perception based on space perception and time perception. Yao et al. (2016) found that acute stress was related to a larger decrease in temporal sensitivity [47]. Based on this finding, it can be inferred that acute psychological stress is related to the improvement of spatial perception. In real life, this alertness or self-defense awareness could make people avoid action in advance when people face dangerous stimuli.

The results also suggest that identifying UD was easier than identifying the other two types of movement. Participants had a shorter reaction time and a higher accuracy when judging UD than when judging CS and UA. Other research has also demonstrated a difference in speed judgments when there was accelerating vs. decelerating movement. For example, people appear to perceive a decelerating object as moving more slowly than an accelerating object [48]. Geesaman and Qian (1998) found that the dot speed of the extended motion seemed to be faster than the dot speed of the rotation under the same dot speed [7]. They also found that the sensitivity of the speed of the translation dot was between that of the expansion and rotation modes. The research found that the perceived rate of movement depends on contrast and at 16 Hz a reduction, in contrast, increases the perceived velocity [5]. Thompson et al. (2006) found that the faster stimulation of movement can appear to move faster than their actual speed at low contrasts [6]. The contrast between UD at the beginning of the movement was larger than that of UA and CS. So, under the condition of UD, the dots’ expansion speed was faster at the beginning and then became slower, which makes it easier to distinguish and thus reduces reaction time.

The current study found that UA could attract people’s attention earlier. The peak latency of the P1 component evoked by the UA was shorter than that evoked by UD. The P1 component is the main component of the early visual information processing stage when the primary features (size, shape, color, etc.), of the visual stimulus, are coded [30]. P1 was related to the inhibition process of the unconscious attentional bias [30]. Early attention occurs when the stimulus information is input into the cerebral cortex center through the visual receptors, and the central sensory analyzer performs primary cognitive processing on the shape and contour of external objects. In the current study, compared with UD, UA was perceived to be slower at the beginning, when most of the dot stimuli were gathered together and might have attracted attention earlier. The outermost dot of the lights showing UD had a fast speed and a large movement range, and people needed to concentrate their attention to distinguish the two types of motion.

The best state of attention control appeared later in the judgment of UD. The peak latency of N2 evoked by UD was significantly longer than that evoked by UA and CS. N2 was closely related to real motion perception, and it appeared about 150–200 ms after the start of movement [49]. The N2 is an ERP component that shows activity during the strategic control process of attention control [50]. The increase in the N2 peak amplitude suggests that attention control was enhanced [51,52]. Increases in anxiety also increase the N2 peak amplitude, suggesting that anxiety activates the cognitive control strategy [53,54]. The outermost dot of the lights showing UD had a large movement range and appeared to have a fast speed. Participants needed to focus on controlling their attention for a long time. So, N2 component appeared later in UD. This study also found that the N2 peak latency while making judgments about UD was shorter under the control condition than that of the stress condition, and the N2 peak latency while making judgments about UA and CS under the control condition were longer than those of the stress condition. The best attention control state would appear later in identifying UD movement, and psychological stress would increase the level of alertness under stress.

The attention control was higher in the judgment of UD. The N2 peak amplitude evoked by the UD was significantly higher than that of UA and CS. Compared with UA and CS, UD was the fastest at the beginning, and the subjects needed better attention control ability to quickly identify the type of speed shown by the dots of light. In addition, the study found that the N2 peak amplitude evoked by judgments made under stress was significantly higher than that evoked under control conditions at CP5. A previous study found that acute psychological stress could induce a psychological state of high arousal and alertness, and a subsequent anxiety reaction [55]. Research has also found that people’s attention range under stress shrinks and affects the efficiency of perceptual processing [45]. At the same time, anxiety and tension would strengthen people’s cognitive control of the attention process [53,54]. In the current study, the attention control ability in the speed perception task was enhanced under stress, which was reflected in the decrease in reaction time and the increase in the N2 peak amplitude.

This study found that there was a larger investment of cognitive resources under stress. The SW mean amplitude during speed perception was significantly lower under the control condition than that under the stress condition. Previous research showed that SW is an ERP component that represents the representation of visual working memory [56]. The long-lasting negative diffuse wave reflects an ERP component activated during the visual–spatial information coding process [57]. SW has been shown to be a component related to the coding stage in memory and its distribution in the brain varies with sensory channels and stimulus types [58]. It can be seen in earlier research that SW is a late component closely related to the information coding of visual space stimuli and the representation of visual working memory. The mean amplitudes of SW are affected by memory load [31]. The current results found that the mean amplitude SW was closely correlated to the consumption of cognitive resources. The lower the SW mean amplitude, the fewer cognitive resources were consumed. Therefore, subjects needed to pay more cognitive resources in the process of discerning the type of motion in the stress condition. In addition, the SW mean amplitude evoked by the perception of UD was lower than that of UA and CS. Referring to [5,6], the contrast of UD was larger than that of UA and CS. Therefore, attention was easily attracted to UD. In addition, the extraction speed of working memory was faster in UD, which needed lower cognitive resources. Similarly, Schlack et al. (2008) found that the perception of an accelerating object’s speed was slower than their perception of a decelerating object’s speed [59]. The results in the current study also showed that people were better at judging deceleration motion, which was reflected in the lower cognitive resources required to identify UD.

Tests of the behavioral data showed that the processing speed was faster under stress than that of the control condition. The ERP data were consistent with this finding. Both the peak amplitude of N2 and the mean amplitude of SW evoked by the speed perception were lower under the control condition than those under the stress condition. Combining the neurophysiological and behavioral results, we can speculate that the shortening of reaction time in the speed perception task under stress was associated with increased N2 activity. It will also make sensory information input and early visual processing more sensitive [60]. Bertsch et al. (2011) found that the speed of perceptual processing increased under stress [46]. Similarly, other research showed that attention control ability improved under stress [53,54].

Based on these studies, it appears that attention control ability might also be improved under stress. In addition, compared with judgments about UA and CS, the reaction time was shorter, the accuracy was higher, and the N2 peak amplitude was longer in judgments of UD. The correlation coefficient between reaction time and N2 peak amplitude was 0.38 (*p* = 0.06) under the UD condition. Schlack et al. (2008) found that people perceived objects faster under a deceleration condition than under an acceleration condition [59]. Their result showed that people had better attention control ability and showed better behavioral performance when judging the speed of UD. In short, there was a connection between behavioral and electrophysiological results. These results supported the hypothesis and explored the electrophysiological mechanism of acute psychological stress on speed perception for the first time. From a practical point of view, acute psychological stress appeared to promote speed perception. Importantly, our findings help to deepen our understanding of the relationship between acute stress and speed perception. There are some limitations in this study, such as low ecological validity and vague stress intensity. In future studies, stress intensity can be divided into multiple levels to further explore the effects of different intensities of acute stress on speed perception.

## 5. Conclusions

The results of the present study suggest several conclusions: It was easier to judge the speed of decelerating objects compared to those showing accelerating motion or constant speed motion. Under acute stress, the reaction speed was faster, and the attention control was improved on the speed perception task together and there appeared to be a greater investment of cognitive resources. The best state of attention control appeared earlier in the judgment of uniform acceleration and constant speed tasks in the stress condition. These findings are best interpreted in light of the link between arousal and attention and support the inverted U-shape hypothesis and the neuromotor interference theory.

## Figures and Tables

**Table 1:** Mean and SD for latency and amplitude of P1, N2. Mean and SD for the mean amplitude of SW.

		Stress Condition			Control Condition		
		CS	UA	UD	CS	UA	UD
P1	Latency (ms)	140.95 ± 12.82	138.12 ± 21.91	143.42 ± 14.23	143.27 ± 13.30	136.44 ± 9.25	148.35 ± 10.05
	Amplitude (μV)	6.31 ± 2.88	6.28 ± 2.43	7.08 ± 2.94	6.49 ± 2.83	6.00 ± 2.63	6.26 ± 2.30
N2	Latency (ms)	194.33 ± 11.97	188.29 ± 12.64	201.45 ± 13.98	194.97 ± 10.34	195.14 ± 13.36	201.45 ± 13.80
	Amplitude (μV)	−3.80 ± 2.05	−3.51 ± 1.84	−4.65 ± 1.79	−3.49 ± 1.88	−3.26 ± 1.67	−4.43 ± 2.04
SW	Amplitude (μV)	−4.77 ± 3.37	−4.48 ± 3.63	−3.22 ± 3.20	−2.27 ± 3.32	−2.24 ± 2.04	−0.27 ± 3.69

## Data Availability

The data used to support the findings of this study are available from the corresponding author upon request. The data are not publicly available due to privacy or ethical restrictions.
